# Decreasing Seroprevalence of Measles Antibodies after Vaccination – Possible Gap in Measles Protection in Adults in the Czech Republic

**DOI:** 10.1371/journal.pone.0170257

**Published:** 2017-01-13

**Authors:** Jan Smetana, Roman Chlibek, Irena Hanovcova, Renata Sosovickova, Libuse Smetanova, Peter Gal, Petr Dite

**Affiliations:** 1 Department of Epidemiology, Faculty of Military Health Sciences, University of Defence, Hradec Kralove, Czech Republic; 2 Department of Rehabilitation, University Hospital, Hradec Kralove, Czech Republic; 3 Military Health Institute, Ceske Budejovice, Czech Republic; 4 Military Health Institute, Brno, Czech Republic; Universidad Nacional de la Plata, ARGENTINA

## Abstract

**Aims:**

In recent years, Europe has recorded an increase in the number of measles outbreaks despite the implementation of vaccination into the National Immunization Programs. The Czech Republic introduced vaccination against measles into National Immunization Program in 1969. The aim of this study was to determine seroprevalence of IgG antibodies against measles in adults.

**Methods:**

Our study was designed as a prospective, multicenter cohort study. Samples of blood were taken from adults aged 18 years and over. Specific IgG antibodies were determined by ELISA method.

**Results:**

A number of 1911 sera samples were obtained. The total seropositivity reached 83.3%, 14.3% of the results were negative and 2.4% were borderline. When comparing the individual age groups, the highest antibody seropositivity (> 96%) was detected in persons aged 50 years and over who were naturally infected in pre-vaccine era. The lowest seropositivity was recorded in the age groups 30–39 years (61.5%), 40–49 years (77.5%) and 18–29 years (81.1%).

**Conclusions:**

A long term high rate of seropositivity persists after natural measles infection. By contrast, it decreases over time after vaccination. Similarly, the concentrations of antibodies in persons with measles history persist for a longer time at a higher level than in vaccinated persons. Our results indicate possible gap in measles protection in adults born after implementation of vaccination into the National Immunization Programs. There are two probable reasons, decrease of measles antibody seropositivity in time after vaccination in setting of limited natural booster and one-dose vaccination schedule used in the first years after implementation.

## Introduction

Measles is a highly contagious viral infectious disease. Measles virus (Morbillivirus) is transmitted from person to person by the airborne route. In the pre-vaccine era more than 90% of people experienced the infection during their childhood. Infants are protected by transplacentally transmitted maternal antibodies for only a few months after birth. Thereafter, all individuals are susceptible to the infection. Natural measles infection results in life-long immunity. Another option to induce a long-term protection is vaccination.

Due to implementation of vaccination against measles into the National Immunization Programs (NIP) of many countries, a significant decline of incidence and the number of deaths from measles has been noted globally. The number of deaths reported worldwide between 2000 and 2010 decreased from 750,000 in 2000 to 197,000 in 2007. About 85% of children worldwide received one dose of the vaccine through the NIP in 2010. The highest incidence of measles is reported in developing countries. But even in the European region of the World Health Organization the incidence of the disease is not currently stable. Between 2010 and 2012, the number of reported cases of measles was in the range from 26,188 to 37,073 per year. The largest epidemics were recorded in countries such as Bulgaria, France, Romania, Ukraine, Germany and the Netherlands. These epidemics were specific by the increase in proportional representation of older children and young adults among the cases of the disease [1]. Various European countries have reported measles outbreaks [2–4]. They have been linked with an increase in the number of susceptible individuals due to low vaccination coverage, which led to a decrease of herd protection. The long term incidence of measles in the Czech Republic was stable. Only a few cases of the disease were reported each year. Of the 87 cases of measles between 2000 and 2010, 36 cases were classified as imported [5]. A significantly increased incidence was recorded in 2014, when 222 cases of measles were reported. This increase was predominantly caused by an epidemic that occurred after import of the infection. During the epidemic a significant spread of the measles virus was described not only in the general population, but also among health care workers, who during the first two months of the onset of the epidemic represented 40% of all affected individuals. Age-specific incidence was the highest among people born between 1970 and 1979 in this outbreak [6].

To ensure individual protection against measles, the World Health Organization recommends the application of two doses of the vaccine to achieve vaccination coverage higher than 95% to ensure herd protection. The essential key group for vaccination against measles is children aged 1 to 4 years. In addition, the importance of health care workers vaccination is being emphasized due to the risk of spreading the infection from health care professionals to patients, as well as from patients to health care professionals [7]. In the Czech Republic mandatory measles vaccination program was implemented into the NIP in 1969 for children at 12 months of life, first in a one-dose schedule, which was changed to a two-dose schedule in 1975. Currently, It uses a live attenuated combined vaccine against measles, mumps and rubella, applied in a two-dose schedule. The first dose is administered to children from the 15^th^ month of life and the second dose 6 to 10 months after the first dose. The assessment of the vaccination coverage in 2009 reported that more than 98% of children born in 2006 have been vaccinated with two doses of vaccine against measles, mumps and rubella, and 0.5% of children had not received even a one dose of the vaccine. However, even localities with vaccination coverage level lower than 95% were recorded [5]. The high level of vaccination coverage has essentially led to elimination of measles.

The children's population in the Czech Republic is currently well protected against measles by post-vaccination immunity. Given the present occurrence of measles epidemics in countries where vaccination against measles has long been a part of the NIP (including the Czech Republic), the question is, how long will the protection against measles induced by vaccination in childhood persist in the adult population. The aim of the presented study was to determine seroprevalence of specific IgG antibodies against measles in the adult population, and to provide data to assess the effectiveness of the National Immunization Program. Another objective was to compare individual age cohorts and to define high-risk groups in the population.

## Materials and Methods

### Study design

The study was designed as a prospective, multicenter, observational cohort study associated with obtaining samples of sera from adults aged 18 years and over and to assess the seroprevalence of specific IgG antibodies against measles. The subject recruitment was conducted with the aim to obtain a representative sample of the population in three centers in geographically different regions of the Czech Republic, during the period from October 2011 to April 2012. Based on an advertising campaign in the media the study participants from general population were recruited on the “first-come, first-served” principle. Blood samples were collected from the study participants. The obtained sera were stored at a temperature of -20°C until the serological examination. The study participants also filled out questionnaires aimed at medical history of vaccination against measles, natural measles infection or contact with measles infected individuals in the last 5 years prior to sample collection. The collected information was used for proper interpretation of the obtained results.

The study protocol was approved by Ethics Committee of University Hospital Hradec Kralove, Czech Republic (201108S15R) and investigation was conducted in accordance with the Declaration of Helsinki. Written informed consent was obtained from all participants before study entry.

### Study population

The randomization of participants ensured uniform representation in individual age categories of 18–29 years (years of birth 1982–1993), 30–39 years (1972–1981), 40–49 years (1962–1971), 50–59 years (1952–1961), 60–69 years (1942–1951), and 70 years and over (1924–1941), corresponding to the population composition of the Czech Republic. The vaccination status of the study population was calculated according to NIP in force at the time of each individual´s immunization based on year of birth. Vaccination against measles in the Czech Republic was introduced in 1969 in a one-dose schedule, which was changed to a two-dose schedule in 1975. It means the age categories 50 years and over included unvaccinated persons who were born in pre-vaccine era, age category 40–49 years included both vaccinated and unvaccinated persons and age categories under 40 years included only vaccinated persons.

### Laboratory methods

The presence of specific IgG antibodies against measles in the obtained sera samples was determined by the ELISA method. We used the commercial RIDASCREEN Measles Virus IgG set (R-Biopharm, Germany), which allows both qualitative and quantitative evaluation of the results. The tests were performed on an automated ELISA TrinLab D2 / DS2 analyzer according to the set manufacturer's user manual. All determinations were performed in duplicate with one batch of the diagnostic set. The results were evaluated qualitatively as positive, negative and borderline. Positive results were evaluated as corresponding to 200 mIU/ml and over, negative under 150 mIU/ml and borderline 150–199 mIU/ml. Sera with a borderline result were subjected to a repeated determination. In case of a borderline result in the repeated determination, the sample was according to the set manufacturer's user manual and recommendations considered as negative. This consequently affected the quantitative evaluation of the results. The concentration of IgG antibodies was calculated from the measured values of optical density, using a four-parameter equation where the constants are calculated by the manufacturer from the 7-point calibration range for each batch of the product and together with the calibration curve are listed in the set's certificate. The results were reported in international units mIU/ml. If the measured optical density of the sample occurred above the upper limit of the calibration curve, then we repeated the determination with a diluted serum. All determinations included commercial negative and two positive controls. The antibodies in vaccinated persons were classified as post-vaccination, while those in unvaccinated persons as post-infection.

### Statistical analysis

The NCSS 9 software was used for statistical analysis of the results. In addition to descriptive statistics, t-test (alternatively nonparametric Mann-Whitney and Kolmogorov-Smirnov tests), nonparametric Kruskal-Wallis one-way analysis of variance followed by Dunn's multiple comparison test with Bonferroni correction to significance level, nonparametric Spearman rank correlation coefficient, chi-square test of independence in contingency tables and Fisher's exact test were used. Results were considered statistically significant at p < 0.05. Appropriate test for statistical analysis was selected based on the distribution of values in each of the compared groups.

## Results

### Demography

A total number of 1911 sera samples were obtained from individuals aged 18–87 years, representing 917 men (48%) and 994 women (52%). Table 1 shows the number of people in individual age groups enrolled in the study. Apart from the group 70 years and over, both the representation of subjects in individual age groups and gender were balanced. The average age of enrolled subjects was 45.5 years, 46.7 years for men and 44.4 years for women. The questionnaire focused on the medical history was completed by 1633 persons (85.4%).

**Table 1:** Demographic characteristics of study subjects.

Age category (years)	Overall		Men		Women	
	n	%	n	%	n	%
**18–29**	387	20,25	173	18,87	214	21,53
**30–39**	374	19,57	181	19,74	193	19,42
**40–49**	356	18,63	144	15,70	212	21,33
**50–59**	307	16,06	153	16,68	154	15,49
**60–69**	350	18,32	178	19,41	172	17,30
**≥70**	137	7,17	88	9,60	49	4,93
**Total**	1911	100,00	917	100,00	994	100,00
**Average age**	45,5		46,7		44,4	

### Serological test results

Table 2 summarizes the results of the qualitative evaluation of IgG antibodies against measles overall and for individual age groups. The total IgG antibody positivity reached 83.3%, 14.3% of the results were negative and 2.4% were repeatedly borderline. There was no statistically significant difference between men and women overall or in the individual age group results.

**Table 2:** Prevalence of IgG antibodies against measles overall, by age group and by sex.

IgG		Positive		Negative		Borderline		Total		p chi-square test
Age (years)	sex	n	%	n	%	n	%	n	%	
**Overall (18–87)**	Men	769	83,86	126	13,74	22	2,40	917	100	0,806
	Women	823	82,80	147	14,79	24	2,41	994	100	
	Total	1592	83,31	273	14,29	46	2,41	1911	100	
**18–29**	Men	135	78,03	30	17,34	8	4,62	173	100	0,280
	Women	179	83,64	30	14,02	5	2,34	214	100	
	Total	314	81,14	60	15,50	13	3,36	387	100	
**30–39**	Men	109	60,22	63	34,81	9	4,97	181	100	0,789
	Women	121	62,69	61	31,61	11	5,70	193	100	
	Total	230	61,50	124	33,16	20	5,35	374	100	
**40–49**	Men	119	82,64	21	14,58	4	2,78	144	100	0,152
	Women	157	74,06	48	22,64	7	3,30	212	100	
	Total	276	77,53	69	19,38	11	3,09	356	100	
**50–59**	Men	144	94,12	8	5,23	1	0,65	153	100	0,179
	Women	151	98,05	3	1,95	0	0,00	154	100	
	Total	295	96,09	11	3,58	1	0,33	307	100	
**60–69**	Men	174	97,75	4	2,25	0	0,00	178	100	0,564
	Women	168	97,67	3	1,74	1	0,58	172	100	
	Total	342	97,70	7	2,00	1	0,29	350	100	
**≥70**	Men	88	100,00	0	0,00	0	0,00	88	100	0,126
	Women	47	95,92	2	4,08	0	0,00	49	100	
	Total	135	98,54	2	1,46	0	0,00	137	100	

**Fig 1:**
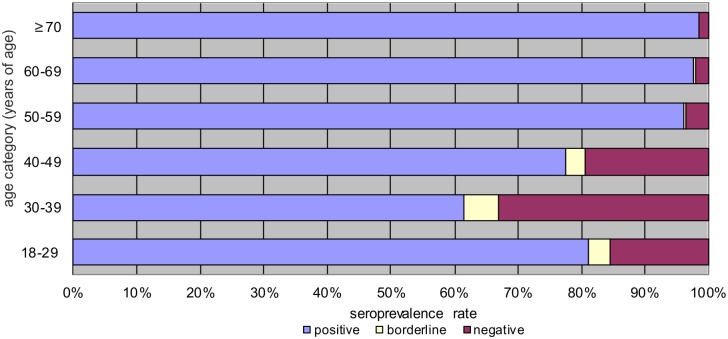
Prevalence of IgG antibodies against measles by age group.

When comparing the individual age groups, the highest seropositivity of IgG antibodies (> 96%) was detected in persons aged 50 years and over. The lowest seropositivity (61.5%) was recorded in the age group 30–39 years, subsequently in the 40–49 years (77.5%) and 18–29 years (81.1%) age groups, respectively (Fig 1). These differences were statistically highly significant. Most of the negative (92.7%) and borderline (95.7%) results were recorded in the three youngest age groups (< 50 years of age), while nearly a half them has occurred in persons aged 30–39 years (45.4%, 43.5% respectively).

**Fig 2:**
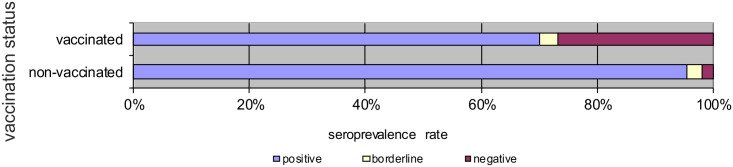
Prevalence of IgG antibodies against measles in group 40–49 years of age.

Persons in the two youngest age groups (18–29 and 30–39 years of age) were vaccinated during their childhood. The age group 40–49 years included persons both vaccinated against measles (249 persons / 69.9%), and unvaccinated (107 persons / 30.1%). Seropositivity of IgG antibodies against measles reached 95.3% in unvaccinated persons, and 69.9% in vaccinated. The difference in seropositivity in vaccinated and unvaccinated individuals was statistically significant (Fig 2).

When comparing overall seroprevalence of IgG antibodies against measles of unvaccinated persons (older persons, before the introduction of nationwide vaccination) and vaccinated persons, seropositivity in unvaccinated persons was found to be 97% and in vaccinated persons only 71%. This difference was statistically highly significant.

**Fig 3:**
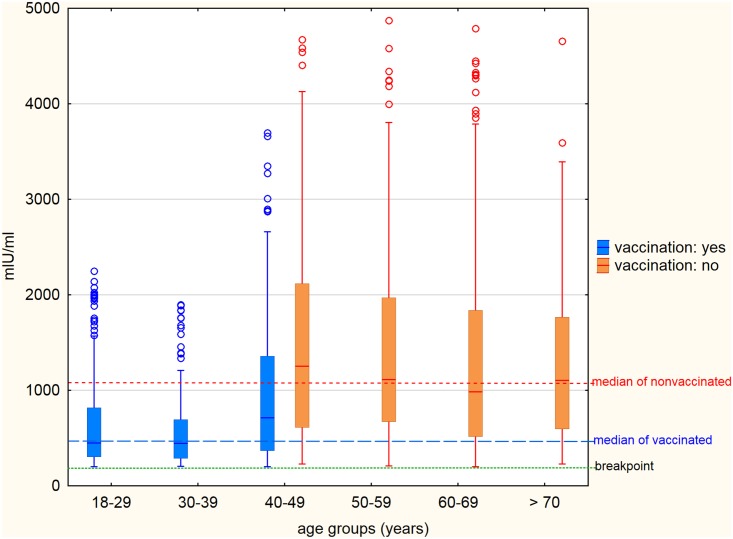
Concentration of IgG antibodies against measles in individual age groups of vaccinated and unvaccinated seropositive persons. The line inside the box shows the median for each group, the lower and the upper edge of the box indicate 25% and 75% percentile, the distance between them (IQR—inter-quartile range) is a measure of the dispersion of values. The ends of the line segments indicate 1.5 times the IQR and values that occur outside these boundaries, are considered outliers.

The presence of IgG antibodies against measles was evaluated quantitatively as well. The comparison of measured concentrations of IgG antibodies against measles in vaccinated and unvaccinated seropositive persons is shown in Fig 3. The medians of concentrations of IgG antibodies were balanced in each individual age group of unvaccinated persons—1250.8 mIU/ml among the 47–49 years, 1114.1 mIU/ml among the 50–59 years, 984.1 mIU/ml among the 60–69 years and 1106.1 mIU/ml among the 70 years and over. By contrast, the detected medians of concentrations of IgG antibodies among vaccinated persons in the lower age groups were statistically significantly lower—446.6 mIU/ml in the age group 18–29 years, 440.4 mIU/ml in the age group 30–39 years and 710.8 mIU/ml in the age group 40–46 years. The concentration of post-infection IgG antibodies in unvaccinated persons expressed as median was 2.3 times greater than the post-vaccination concentration in vaccinated persons (1078.8 mIU/ml vs. 477.8 mIU/ml).

**Fig 4:**
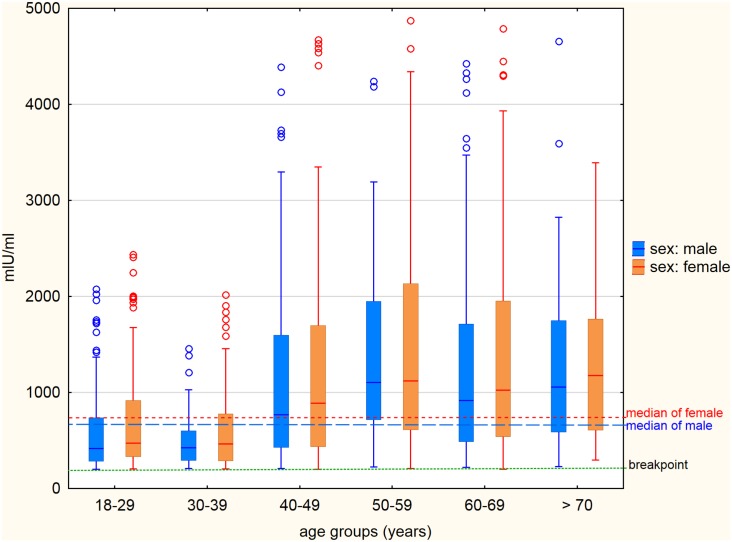
Concentration of IgG antibodies against measles in individual age groups in seropositive men and women. The line inside the box shows the median for each group, the lower and the upper edge of the box indicates 25% and 75% percentile, the distance between them (IQR—inter-quartile range) is a measure of the dispersion of values. The ends of the line segments indicate 1.5 times the IQR and values that occur outside these boundaries, are considered outliers.

The determined concentrations of IgG antibodies in seropositive persons in individual age groups for men and women are described in Fig 4. No statistically significant difference between men and women was found in any age group. The median of concentrations of IgG in seropositive men regardless of age was 735.8 mIU/ml, while in seropositive women 745.1 mIU/ml. However, the determined concentrations of IgG antibodies in men and women in higher age groups (40 years and over) were statistically significantly higher compared to age groups of 18–29 and 30–39 years. The median of concentrations of IgG antibodies in seropositive persons were 1040.0 mIU/ml for unvaccinated men, and 1103.3 mIU/ml for unvaccinated women. For vaccinated seropositive men the median of concentrations of IgG antibodies reached 455.5 mIU/ml and for vaccinated seropositive women 501.3 mIU/ml.

Within the framework of the questionnaire survey, 59.9% of unvaccinated individuals reported natural infection of measles, 15.0% reported no measles infection and 25.1% of subjects did not know, whether they ever had the disease. Seropositivity of IgG antibodies in unvaccinated persons with history of natural infection reached 96.9%, in persons with alleged no measles history 96.5% and in persons with insufficient knowledge of their measles history 97.4% (not included in statistical assessment). The difference in results between the groups of unvaccinated persons with history of natural infection and persons with alleged no measles history was not statistically significant (Table 3). Similarly, a statistically significant difference was not detected even in the concentration levels of IgG antibodies against measles in seropositive unvaccinated persons between these two groups (Table 4).

**Table 3:** Prevalence of IgG antibodies against measles in unvaccinated cohort in relation to measles history.

Measles—disease	Positive		Negative		Borderline		Total		p chi-square test
	n	%	n	%	n	%	n	%	
**Yes**	441	96,92	12	2,64	2	0,44	455	100	0,688
**No**	110	96,49	4	3,51	0	0,00	114	100	
**Total**	551	96,84	16	2,81	2	0,35	569	100	

**Table 4:** Concentration of IgG antibodies against measles in unvaccinated persons in relation to the disease history.

Measles—disease	n	mIU/ml					p Kolmogorov-Smirnov test
		median	95% LCL	95% UCL	min	max	
**Yes**	441	1117,7	1034,6	1247,6	203,8	24014,0	0,397
**No**	110	1076,4	824,1	1213,4	224,5	14033,0	

LCL—lower confidence limit; UCL—upper confidence limit

## Discussion

Over the past decade, except for 2014, only isolated cases of measles have been reported in the Czech Republic. Such epidemiological situation can be connected with high vaccination coverage against measles, which in the Czech Republic, over the long term, exceeds 95% [5]. Nationwide mandatory vaccination in the Czech Republic was introduced in 1969 in a one-dose schedule, which was changed to a two-dose schedule in 1975. Individuals vaccinated with one dose of the vaccine could have been revaccinated within the vaccination campaigns provided for 6–15 years old children [8]. In other European countries vaccination against measles was implemented into the NIP during a similar timeframe. Given the unstable epidemiological situation of measles in Europe, we are faced with a question of the persistence of the protection in adulthood after vaccination during childhood.

### Natural infection provides longer term protection than vaccination

In presented study we recorded the overall seropositivity of the specific IgG antibodies against measles 83.3% for persons aged 18 years and over. A 14.3% of the tested samples were seronegative and 2.4% were borderline. Analysis of the results in the individual age groups, respectively the analysis based on the vaccination status against measles showed higher susceptibility to measles in adults vaccinated in childhood compared to unvaccinated with history of natural infection. Seropositivity > 96% has been detected within the higher age groups not vaccinated against measles in childhood. High seropositivity of antibodies against measles in persons born during the pre-vaccination era was confirmed in concordance with our results by the National Serological Survey performed in the Czech Republic in 2013. Similar findings have been confirmed in other countries [9]. This high seropositivity in our study also corresponds with similar findings, that the immune response, after natural infection, is a longer term and on a higher level than post-vaccinated [10].

### Post vaccination immunity decreases in time

The lowest seropositivity in our study was detected in the age group of 30–39 years (61.5%). This age group was formed by vaccinated persons, but partially those who were vaccinated only one dose of vaccine during the first years of vaccination implementation into the NIP. The second lowest seropositivity was detected in the age group of 40–49 years (77.5%). This group was formed by both unvaccinated persons (30%) and vaccinated, most likely with one dose of the vaccine (70%). The seropositivity of IgG antibodies in this group was 95.3% and 69.9% respectively. Seropositivity of 81.1% was detected in the age group 18–29 years. The decrease of immune response over time corresponds to previous findings that point to lower titres of antibodies against measles in era of the absence of a natural contact with measles [11]. Our results suggest that the one-dose schedule results in lower immune response than the two-dose schedule. Simultaneously, an increase of seronegative individuals among the vaccinees from the time of vaccination can be observed. In our study seronegative persons accounted 15.5% in the age group 18–29 years and in the age group 30–39 years even 33.2%. The serological survey conducted in the Czech Republic in 2013 confirmed trend of seropositivity decrease in the age group 30–44 years (30–34 years 86% seropositivity, 35–39 years 83% and 40–44 years 77%), although the results of our study describes an even greater decrease in seroprevalence of antibodies among adults. Similar trends have also been described for the child population [12].

Such findings suggest a possible gap in protection of population against measles. A possible confirmation of such a gap may be the measles outbreak, which occurred in one region of the Czech Republic in 2014, during which 186 laboratory confirmed cases were recorded [13]. The most affected age group was formed by persons aged 34–44 years, which corresponds with our finding of the lowest seropositivity of IgG antibodies [6]. The reason of higher incidence of measles in this age group is unknown. It is possible to debate, for example, the quality and handling of the vaccine used, the use of a one-dose vaccination schedule, primary or secondary vaccine failure and the waning immunity after vaccination. The outbreak also affected a high percentage of health care workers (37%). This illustrates the fact that in the era of low incidence of the disease, a high risk of nosocomial spread exists due to a lesser experience of health care workers with the disease. While infected, health care workers may be subsequently the source of spreading measles to susceptible individuals both within health care facilities and within the general population [14–18]. For this reason it is necessary to ensure sufficient education of health care workers in matters of epidemiology, transmission, clinical manifestations and measles prevention.

During the quantitative evaluation of IgG antibody concentration levels within our study, statistically significantly higher concentration levels of antibodies were detected in people in higher age groups (unvaccinated) than among younger people (vaccinated). Similar findings of higher antibody levels in people with history of natural measles infection than in vaccinated individuals were also reported in other European countries [19–23]. Clinical and epidemiological impact of this difference is not clearly known.

### Passive immunity may be compromised in newborns of vaccinated mothers

Low levels of antibody concentrations in women of fertile age described in our study may also affect the quality of measles protection of unvaccinated newborns and infants. Infant protection is provided via transplacental transmission of antibodies in case that the mother has been vaccinated or had measles during life. The protection is provided only for the period of approximately 12 months after birth. Some studies, however, demonstrate earlier disappearance of maternal antibodies, which may lead to earlier susceptibility to measles in infants in their first year of life and to eventual consideration of timely application of the first dose of the vaccine [24–26]. From this point of view it is possible pose a question about the level of protection against measles in children of mothers, who have low seropositivity levels or low antibody concentrations in the serum. Especially, if we take into account the increasing average age of pregnant women. In children of mothers with a history of measles higher antibody titers were reported than in children of vaccinated mothers without a history of measles [19,27–29]. Timely vaccination of infants is therefore important both for the induction of protection among vaccinated children, but also from the perspective of seronegative adults, for whom the children can be a source of infection.

### Vaccination in travelers

The low seroprevalence of IgG antibodies against measles in adults should lead travelers visiting areas with a high incidence of measles to an assessment of the vaccination status and to consider a recommendation for revaccination.

## Conclusions

Our results confirm the long-term persistence of high seropositivity rate after natural measles infection. By contrast, seropositivity after vaccination decreases over time. Similarly, the concentrations of specific antibodies in persons with history of measles persist for a longer time period and at a higher level than in vaccinated persons. It shows that natural infection provides better protection than vaccination. Our study indicate possible gap in measles protection in adults born after implementation of vaccination into the NIP. Achieved results should start a discussion about real level of measles susceptibility in adults in the Czech Republic, particularly in persons born during 1970–1980. Because of persistence of immunity, measles might therefore become a disease that can occur more often even in countries with high levels of vaccination coverage, such as the Czech Republic, not only in unvaccinated children, but especially in the adult population.
